# β-Endorphin Induction by Psychological Stress Promotes Leydig Cell Apoptosis through p38 MAPK Pathway in Male Rats

**DOI:** 10.3390/cells8101265

**Published:** 2019-10-16

**Authors:** Xiaofan Xiong, Lingyu Zhang, Meiyang Fan, Lin Han, Qiuhua Wu, Siyuan Liu, Jiyu Miao, Liying Liu, Xiaofei Wang, Bo Guo, Dongdong Tong, Lei Ni, Juan Yang, Chen Huang

**Affiliations:** 1 Department of Cell Biology and Genetics, School of Basic Medical Sciences, Xi’an Jiaotong University Health Science Center, Xi’an 710061, China; xxf06152458@stu.xjtu.edu.cn (X.X.); zly377619125@stu.xjtu.edu.cn (L.Z.); pursuit@stu.xjtu.edu.cn (M.F.); hanlin2016@stu.xjtu.edu.cn (L.H.); klva2019@163.com (Q.W.); liusyuanshen@stu.xjtu.edu.cn (S.L.); miaojiyu@stu.xjtu.edu.cn (J.M.); bo_guo@xjtu.edu.cn (B.G.); tongdd@xjtu.edu.cn (D.T.); nilei@xjtu.edu.cn (L.N.); 2 Key Laboratory of Environment and Genes Related to Diseases (Xi’an Jiaotong University), Ministry of Education of China, Xi’an 710061, China; llying@xjtu.edu.cn (L.L.); wxiaofei@xjtu.edu.cn (X.W.)

**Keywords:** psychological stress, β-endorphin, sperm quality, Leydig cells, p38 MAPK

## Abstract

Psychological stress (PS) disturbs the reproductive endocrine system and promotes male infertility, but the underlying pathogenic mechanisms have not been extensively studied. This study aimed to uncover the mechanisms of PS-induced male reproductive related abnormalities subjected to a ‘terrified sound’ exposure. Male rats subjected to PS displayed slow growth, decreased sperm quality, abnormal levels of the reproductive endocrine hormones, decreased expression of the reproductive-related proteins androgen-binding protein (ABP) and bromodomain-containing protein (BRDT), increased apoptosis in the testis, and accompanied by elevated levels of β-endorphin (β-EP). These effects were reversed by naloxone. Furthermore, PS-induced β-EP could promote mu opioid receptor (MOR) activation and ensure intracellular p38 MAPK phosphorylation and then lead to Leydig cells (LCs) apoptosis. The current result showed that β-EP was a key factor to PS-induced male infertility.

## 1. Introduction

Male infertility can lower self-esteem and disrupt family harmony. Unfortunately, this condition may be difficult to treat because the underlying cause is unknown in the majority (about 70%) of cases [1]. Psychological stress (PS) has been reported that it could lead to a significant decrease in plasma testosterone (T) levels [2,3], thereby reducing sperm quantity and vitality [4]. Psychotherapy has been shown to improve sperm quality in patients with infertility caused by PS [5]. Moreover, animal research has demonstrated that stress can disrupt the function of the hypothalamic−pituitary−testis axis (HPT axis) by reducing the release of luteinizing hormone (LH) and follicle stimulating hormone (FSH) [6,7], resulting in apoptosis of testicular cells [8].

β-endorphin (β-EP) may be a critical mediator of male infertility due to PS. It is synthesized and secreted in both central nervous system structures controlling sexual function, notably the pituitary and hypothalamus, as well as in the testis, including LCs and sperm [9]. Systemic levels increase at least five- to tenfold under stress [10,11,12,13] and elevation is strongly associated with hyperactivity of the hypothalamic−pituitary−adrenal axis (HPA axis). Under stress, enhanced corticotropin releasing hormone (CRH) production by the hypothalamus accelerates β-EP and adrenocorticotropic hormone (ACTH) synthesis and release by the pituitary. Both animal and clinical studies have provided direct evidence that excessive β-EP can disrupt the male reproductive endocrine system through preferential action at different receptor sites, and β-EP production was controlled by gonadotropins directly and modulated by steroids [14,15]. For instance, heroin addiction leads to sexual dysfunction characterized by reduced sperm quantity and vigor [16], and long-term morphine administration to male rats inhibits T secretion [14]. Elevated β-EP is also associated with weak spermatozoa and low sperm count in male infertility patients [9]. At the molecular level, β-EP inhibits the pituitary secretion of FSH and LH by blocking hypothalamic release of gonadotropin releasing hormone (GnRH), thereby inhibiting gonadal T secretion and reducing sperm count and vitality [17,18]. Mammalian spermatogenesis depends on tropic support of spermatogenic cells, testicular Sertoli cells, and LCs by gonadotropins. LCs produce T in response to LH secreted by basal cells of the anterior pituitary. Systemic T supports the maintenance of secondary sexual characteristics while local T promotes spermatogenesis by binding to androgen receptors. Reduced LH secretion also induces β-EP synthesis by LCs, further inhibiting T secretion and sperm production. Inhibition of steroid biosynthesis also significantly increased β-EP levels, and the synthesis and regulation of β-EP in LCs have been reported in several previous studies [19,20,21]. However, its mechanism is still unclear.

Here, we aimed to comprehensively reveal a pathway for stress-induced reproductive damage through a PS animal model. The analysis of behavior, sperm quality, endocrine hormones, testicular germ cell apoptosis, reproductive-related protein expressions, β-EP and its receptor expressions were used to clarify whether β-EP was related to male reproductive damage. And then, cell molecular experiments were conducted to investigate the signaling pathway of β-EP promoting apoptosis.

## 2. Materials and Methods

### 2.1. Animals

Adult male Sprague-Dawley (SD) rats (8–10 weeks, 300 ± 10 g) were obtained from the Medical Experimental Animal Centre of Xi’an Jiaotong University (Shaanxi Province, China). Animals were housed 5 per cage under controlled temperature (23 ± 2 °C), controlled humidity (50%), and a 12 h/12 h light/dark cycle with ad libitum access to food and water. All animal care and experimental protocols where approved by the Animal Care Committee of Xi’an Jiaotong University (No. XJTULAC2019-1206).

### 2.2. Psychological Stress Model

Rats were adapted to the laboratory environment for 7 days before grouping and experimentation. Thirty male rats were randomly assigned to the PS group and the control group (n = 15/group). Rats in the PS group were exposed to a ‘terrified sound’ stress at 45−60 dB (as measured by a SL-5800 sound meter, Landtek Instruments, Guangzhou, China) for 3 h in the morning and 3 h in the afternoon for 21 consecutive days delivered through loudspeakers (Edifier R1000TC speakers, Beijing, China) placed 50-cm above each side of the cage. Animals in the control group were exposed to the same room conditions but without sound exposure. The sound-induced stress protocol was described in detail previously [22,23,24]. The experiment schedule is provided in Figure S1.

### 2.3. Intervention Group Processing

Four intervention groups (n = 15/group) were established: a control group administered saline (1 mL/time) by intraperitoneal (i.p.) injection, a PS group i.p. injected with saline following the same regimen, a β-EP injection group (0.2 mg/kg), and a PS plus naloxone injection group (3 mg/kg). All injections were given every second day. Drug doses were set according to a previous study [25].

### 2.4. Sucrose Preference Test

The sucrose preference test was conducted as a measure of stress-induced anhedonia in a quiet room with low lighting. Briefly, rats were housed singly without water for 24 h. A bottle of 1% sucrose water and a bottle of pure distilled water were placed in the cage side-by-side in random order and the volumes consumed over 24 h measured according to container weight changes. Sugar water preference index (%) was calculated as sugar water consumption/(sugar consumption + pure water consumption) × 100%.

### 2.5. Collection of Serum, Testis, and Epididymis

To test the effects of stress on reproductive function, five rats were randomly selected from each group, anesthetized using 10% chloral hydrate (25–30 mL/kg body weight), and sacrificed by cervical dislocation for blood and tissue sampling. Blood samples were drawn for hormone measurement, and tissue samples were obtained for weighing, sperm count and mobility tests, and histology. Briefly, blood samples were clotted for 2 h in serum separator tubes at room temperature and centrifuged at 4 °C for 20 min at 1500× g. The serum was quickly separated and stored at −20 °C until hormone measurements (described below). For tissue sampling, the abdominal hair was shaved, the skin disinfected with 75% ethanol, a straight incision made along the midline of the pelvic cavity, and the scrotal skin opened. Testes were separated from cauda epididymis and cleared of connective tissue. The isolated epididymis and testicles were immediately placed in an electronic microbalance and weighed. The testicles were then fixed with 4% paraformaldehyde solution for paraffin embedding and sectioning (detailed below).

### 2.6. Sperm Count and Mobility Assays

The cauda epididymis was cut into three segments using ophthalmic scissors. Sperm were harvested and transferred to 24-well cell culture plates with 1.0 mL of 37 °C pre-warmed physiological saline and gently shaken at 100× *g* for 30 min. Sperm quality was analyzed using a Weili WLJY-9000 color sperm quality detection system, an Olympus microscope, and a Makler counting plate (Beijing, China). According to the World Health Organization (WHO) Semen Analysis and Processing Experiment Manual, 4 μL of the sperm suspension at room temperature (25 °C) was added to the Makler plate counting area, and individual spermatozoa counted under light microscopy.

### 2.7. ELISA Assay

Serum levels of cortisol (CORT), GnRH, CRH, ACTH, FSH, LH, T, and β-EP were measured by double-antibody sandwich enzyme-linked immunosorbent assay (ELISA) kits (Elisa Biotech, Shanghai, China). Briefly, standards with known hormone concentrations and plasma samples were added to wells pre-coated with plasma serine protease inhibitor, followed by addition of the target antibody and HRP-conjugated secondary antibody. After incubation and washing, HRP substrate was added, followed by stopping solution. The optical density (OD) at 450 nm was measured within 15 min using a microtiter plate reader (FLUOstar Omega, BMG LABTECH GmbH, Germany).

### 2.8. Immunohistochemistry

Testicular tissue sections (5 µm) were deparaffinized using xylene, rehydrated in gradient ethanol (100%, 95%, 90%, 80%), incubated in endogenous peroxidase inhibitor to eliminate endogenous peroxidase activity, and then blocked with goat serum working solution (all at room temperature). Antibody was then added drop-wise to the sections. Sections were placed in a wet box and incubated at 4 °C overnight. The next day, secondary antibody was added for 15 min at 37 °C. Immunolabeling was revealed by 3, 3´-diaminobenzidine (ZSGB-BIO, Beijing, China). Sections were counterstained with hematoxylin, dehydrated in graded ethanol (80%, 90%, 95%, 100%), made transparent using xylene, and sealed with neutral gum for further analysis.

### 2.9. Evaluation of Immunohistochemical Staining

Staining intensity and staining percentage of testicular tissue sections were semi-quantitatively analyzed used H-score [26]. There were four grades of intensity staining: no (0 point), weak (1 point), medium (2 points), and strong (3 points). Five non-overlapping fields were randomly selected for each slice, calculated the staining percentage and scored the staining intensity. H-score score = Σ (I × PC). I and PC represented the positive intensity (0–3), and the percentage of positive cells (0–100), respectively. The final H-score ranging from 0 to 300.

### 2.10. HE Staining

For hematoxylin and eosin (HE) staining, paraffin sections were dewaxed twice in xylene, rehydrated in gradient ethanol (100%, 95%, 90%, 80%), rinsed in pure water and then successively immersed in hematoxylin for 3−10 min, 1% hydrochloric acid alcohol differentiation solution for about 5 s, warm water for 10−30 s (until the section turned blue), and in eosin for 1−3 min. Sections were then dehydrated in gradient ethanol (80%, 90%, 95%, 100%), made transparent with xylene, and sealed with neutral gum.

### 2.11. TM3 Cell Culture and Treatment

Mouse TM3 cells, which have characteristics similar to LCs such as LH response, were obtained from Zhongqiaoxinzhou Biotech (Shanghai, China). Cells were grown in Dulbecco’s modified Eagles medium (DMEM)/F12 supplemented with 5% horse serum, 2.5% fetal bovine serum, and 1% antibiotic solution (100 U/ml penicillin and 100 mg/ml streptomycin sulfate) under 5% CO_2_ at 37 °C. Cells were subcultured for three generations after recovery prior to experiments. TM3 cells were exposed to β-EP (1−100 nM) and (or) naloxone (1−100 µM) for 48 h and then evaluated for viability, apoptosis rate, and protein expression as described below.

### 2.12. MTT Assay

Cell viability was estimated using the 3-4,5-dimethylthiazol-2-yl-2, 5-diphenyltetrazolium bromide (MTT) assay. Briefly, TM3 cells were cultured in 96-well plates (1500 cells/well) with five technical replicates per plate and treated with β-EP and (or) naloxone for 24, 48, or 72 h. For estimation of viable cell number, MTT solution was added to the treated cells and incubated for 4 h at 37 °C. The supernatant was discarded and formazan produced by viable cells dissolved in dimethyl sulfoxide. Absorbance was measured at 492 nm using a FLUOstar Omega plate reader (BMG LABTECH GmbH, Germany).

### 2.13. TUNEL Assay

Testicular germ cell apoptosis was detected by the DeadEnd™ Fluorometric TUNEL System (G3250, Promega, Madison, WI, USA). Paraffin-embedded tissue sections were dewaxed in xylene, rehydrated in gradient ethanol (100%, 95%, 85%, 70%, 50%), washed in 0.85% NaCl. Cells were then permeabilized with Triton® X-100 and tissue sections with proteinase K. Samples were wash in PBS, incubated in kit Equilibration Buffer, and covered with plastic coverslips. Slides were then immersed in kit incubation buffer (Equilibration Buffer, Nucleotide Mix, rTdT Enzyme) at 37 °C for 1 h under darkness. Coverslips were then removed and the slides dipped in 2× saline sodium-citrate (SSC) buffer. DNase I was used to generate strand breaks in DNA to provide a positive TUNEL reaction control. Control incubated buffer without rTdT Enzyme have been prepared for negative controls, so the strand breaks in DNA cannot be labeled by 12-dUTP. Nucleus of samples were counterstained with DAPI and sealed under mounting medium for fluorescence microscopic analysis. The numbers of green fluorescent apoptotic cells (fluorescein-12-dUTP) were counted on the blue DAPI background.

### 2.14. Quantitation of Apoptosis by Flow Cytometry

For quantitation of apoptosis rate, TM3 cells were harvested 48 h after the indicated treatment, washed twice with PBS, stained using the Annexin V-FITC/PI Apoptosis Detection kit (7sea biotech, Shanghai, China) and counted by flow cytometry.

### 2.15. Western Blotting

For total proteins, tissue or cell samples were homogenized in RIPA buffer (FDbio Science, Shanghai, China) supplemented with protease inhibitors, centrifuged at 6000× *g* for 10 min, 4 °C. For cytosolic proteins, cells were collected and dried, Cytoplasmic Extraction Reagent (PIONEER, Shanghai, China) was added with vortex mix and reacted on ice, leaving the supernatant after centrifugation. Lysate protein content was quantified using a BCA Protein Assay Kit (GenStar, Shanghai, China). Equal amounts of 20 µg protein per gel lane were separated on 8%−10% polyacrylamide gels, then transferred to PVDF membranes. Membranes were blocked for 1 hour at room temperature with 5% skim milk in Tris-buffered saline plus Tween-20 (TBST), and then incubated with primary antibody overnight at 4 °C. And then placed at room temperature for 30 min, washed three times with TBST for 10 minutes, incubated with the corresponding secondary antibodies for 2 h at room temperature, washed again. Finally, chemiluminescence detection were visualized using enhanced chemiluminescence reagent (DiNing Biotechnology, Beijing, China). All individual proteins was detected under its loading controls on the whole membrane as Figure S6. Antibodies such as ABP, BRDT, p38 MAPK, Bax, Bcl2, caspase3, etc. used in this study were all listed in Table S1.

### 2.16. siRNA Synthesis and Transfection

Short interfering (si)RNA sequences (in Table S2) for MOR and p38 MAPK were pre-designed and synthesized by GenePharma Corporation (Shanghai, China). Scramble siRNA was used as a negative control (NC-siRNA). The siRNAs were transiently transfected into TM3 cells using jetPRIME Transfection Reagent (Polyplus Transfection, France) after culturing for 24 h on cell culture plates.

### 2.17. qRT-PCR Analysis

Total RNA was isolated using Trizol Reagent (Genestar, Shanghai, China) and quantified by a NanoDrop spectrophotometer (Thermo, USA). Quantitative real-time PCR (qRT-PCR) was performed using the FTC-3000P Real-Time Quantitative Thermal Cycler (Funglyn Biotech, Canada) and analyzed by the 2^−ΔΔCt^ method. The PrimeScript RT Reagent Kit and the SYBR Premix Ex Taq II Kit (Genestar, Shanghai, China) were used for qRT-PCR reactions, and target gene expression was normalized to GAPDH expression. The primers were synthesized by T_SING_K_E_ (Xi’an, China). The sequences used for MOR, p38 MAPK, and GAPDH are presented in S3 Table.

### 2.18. Statistical Analysis

All experiments were performed with at least three independent replicates. Statistical analysis and figure construction were performed using GraphPad Prism version 5.01. All data are expressed as mean ± standard error of the mean (SEM). Student’s *t*-test and ANOVA have been used in data analysis, p < 0.05 was considered statistically significant (*) and p < 0.01 as extremely significant (**). Immunofluorescence image integration, western blot densitometry, and immunochemistry grayscale measurements were conducted using Image J 2.0 (NIH).

## 3. Results

### 3.1. PS Lead to Male Reproductive Related Dysfunction

**Figure 1:**
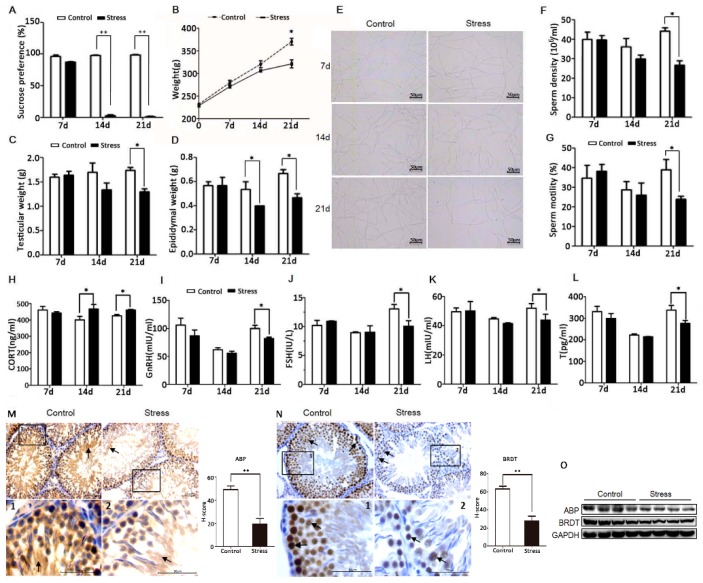
PS impairs reproductive function in male rats. (**A**) Reduced sucrose preference of PS rats vs. controls. (**B**–**D**) Reduced body, testicular, and epididymis weights of PS rats vs. controls. (**E**) Temporary slide microscopy revealing lower sperm count in the cauda epididymis of PS rats vs. controls. Bar = 50 µm. (**F**,**G**) Reduced sperm motility and density in the cauda epididymis of PS rats. Altered serum CORT (**H**), GnRH (**I**), FSH (**J**), LH (**K**), and T (**L**) concentrations in PS rats vs. controls. Reduced expression levels of ABP in the lumen of the seminiferous tubules (**M**), and BRDT in the nucleus of germ cells (**N**) by PS as determined by immunochemistry (black arrow). Testicular tissue sections that are partially magnified are displayed. Bar = 50 μm. The H-score demonstrated that ABP and BRDT were significantly lower in the stress group vs. the control group. (**O**) Reduced ABP and BRDT in PS rat testis as measured by western blotting. All measures in this and subsequent figures were conducted on day 21 (21d) of PS unless otherwise indicated.

**Figure 2:**
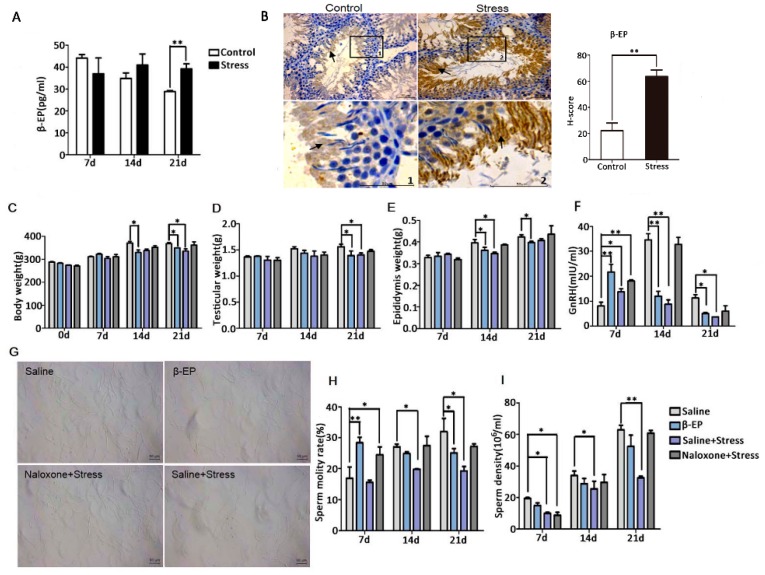
Reproductive dysfunction is associated with PS-induced β-EP elevation. (**A**) Elevated serum β-EP concentration in PS rats vs. controls as measured by ELISA. (**B**) Elevated β-EP in extracellular region in the lumen of the seminiferous tubules (black arrow) in testicular tissue of PS rats as measured by immunohistochemistry. Testicular tissue sections and partially magnify were displayed. Bar = 50 μm. The H-score demonstrated that β-EP was significantly higher in the stress group vs. the control group. (**C**–**E**) Reduced body, testicular, and epididymis weights of rats injected with β-EP. (**F**) Reduced serum GnRH level in β-EP-injected rats. (**G**) Temporary slide microscopy showing reduced sperm count in cauda epididymis of β-EP-injected rats. Bar = 50 μm. (**H**,**I**) Reduced sperm motility and density in β-EP-injected rats. All effects were reversed by naloxone.

**Figure 3:**
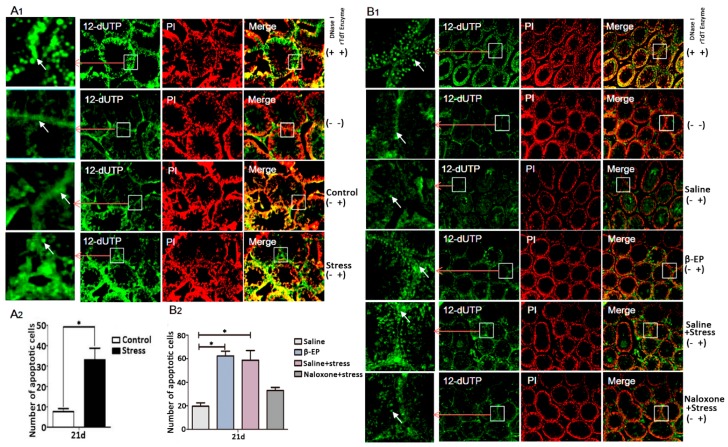
PS induces cell apoptosis in testis. (**A1**,**A2**) PS increased apoptosis of LCs and spermatogenic cells in testis of PS rats vs. controls at day 21 as evidenced by TUNEL staining. Apoptotic cells were shown in green positive areas (indicated by white arrows). The number of apoptotic cells was counted using ImageJ 2.0. (**B1**,**B2**) β-EP injection increased and naloxone reversed testicular cell apoptosis. Whether added DNase I / rTdT Enzyme or not was indicated by “+” and “−”.

To establish a chronic PS model, adult male SD rats were exposed to a ‘terrified sound’ stress in two daily 3-h sessions for 21 consecutive days. At the beginning of the stress period, rats exhibited nervousness and irritability. By the 14th day, they showed decreased activity (lethargy), dull coat color, and slower weight gain than controls. The sucrose preference tests revealed significant anhedonia in PS group rats (Figure 1A), suggesting the onset of a stress-induced depression-like phenotype. In addition to body weight, both testis and epididymis weights were significantly reduced compared to the control group at day 21 (Figure 1B–D). Sperm motility and sperm count were also significantly lower than controls on day 21 (Figure 1E–G). Serum concentrations of the neuroendocrine hormones CRH and ACTH and of the adrenocorticotropic hormone corticosterone (CORT) were abnormal on day 21 (Figure S2A,B; Figure 1H). These abnormalities were accompanied by reduced serum concentrations of the reproductive endocrine hormones GnRH, LH, FSH, and T on 21 d (Figure 1I–L, Figure 2F). Hematoxylin-eosin staining of testis sections also revealed significantly reduced spermatogenic cell numbers in the PS group by day 21 (Figure S2C), while the number of apoptotic cells in the testis was significantly higher (Figure 3A). Given the magnitude of these changes, all subsequent studies were conducted on day 21. Immunohistochemistry (Figure 1M,N) and western blot analysis (Figure 1L) revealed significantly reduced expression of ABP and BRDT in the testis of PS rats. In summary, these results suggest that long-term PS inhibits the function of the HPT axis in male rats, leading to abnormal spermatogenesis.

### 3.2. PS Induces β-EP Secretion in Male Rats

**Figure 4:**
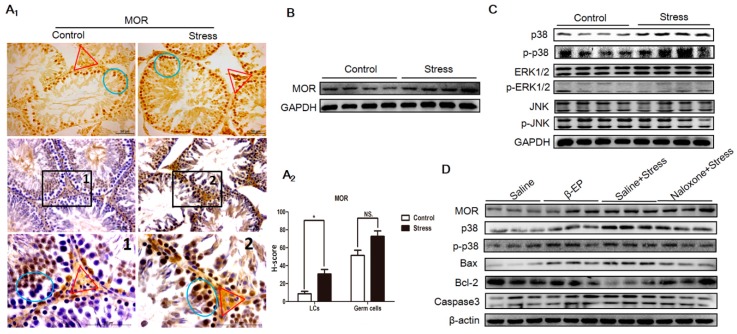
PS-induced apoptosis is associated with overexpression of MORs in testes and activation of the p38 MAPK pathway. (**A1**) Elevated expression of MOR in LCs (red triangle) and germ cells (blue circle in the seminiferous tubule) as evidenced by immunohistochemistry. DAB (Diaminobenzidine) detection, hematoxylin counterstaining, and partially magnify after counterstaining were showed in testicular tissue sections. (**A2**) H-scores of both LCs and germ cells demonstrated that MOR in LCs were significantly higher in the stress group vs. the control group. (**B**) Elevated MOR expression in testes as indicated by Western blotting. (**C**) Selective phospho-activation of p38 MAPK but not ERK1/2 or JNK in the testis of PS rats as indicated by Western blotting. (**D**) Elevated MOR, p38 MAPK, Bax, and caspase 3 expression in the testis of β-EP-injected rats and reversal by naloxone.

β-EP is elevated under stress, and previous studies have linked β-EP elevation to reproductive dysfunction and reproductive organ damage [15−19]. Serum β-EP was also significantly higher in PS rats compared to controls (Figure 2A), and this elevation was accompanied by a significant increase in β-EP-immunopositive regions in the testis (Figure 2B). These immunopositive cells were mainly distributed in the seminiferous tubules around the sperm. In addition, expression levels of the three β-EP receptors (mu [MOR], delta [DOR], and kappa [KOR]) were upregulated in the testis of PS rats (Figure 4A,B, Figure S3). The MOR-positive region was mainly located between spermatogenic cells and on LCs, while DOR- and KOR-positive regions were mainly located in the local organs of the seminiferous tubules, with a few around LCs. Thus, long-term PS may elevate β-EP signaling in LCs through MORs.

### 3.3. β-EP Participate in PS-Induced Sperm Reduction

To confirm if β-EP elevation is directly involved in male reproduction, we examined the effects of β-EP administration with or without the opioid receptor antagonist naloxone. Injection of β-EP alone reduced whole body weight, testis weight, epididymis weight (Figure 2C–E), and serum GnRH (Figure 2F), consistent with the PS model, while naloxone co-treatment reversed these changes to the levels of saline-injected controls. The elevated serum levels of CRH, ACTH, and CORT (Figure S3D–F), the low sperm density and viability (Figure 2G–I), and greater apoptosis rate in testes (Figure 3B) of PS rats were also induced by β-EP injection and reversed by naloxone. Collectively, these findings suggest that stress-induced β-EP inhibits the secretion of GnRH, thereby reducing secretion of FSH and LH by the pituitary gland, which in turn reduces the number and vitality of sperm.

### 3.4. PS Induces Apoptosis in Testicular LCs of Male Rats

In male testis, reduced LH secretion induces β-EP synthesis in LCs, but the pathogenic pathway leading to reproductive dysfunction is still unclear. Most apoptotic cells (Figure 3A,B) and most MOR-positive regions colocalized with LCs and germ cells (Figure 4A,B) in the testis, strongly suggesting that β-EP can directly induce LC apoptosis via upregulation and stimulation of MORs.

We first examined the potential contribution of MAPK signaling to LC apoptosis by extracting total protein from the testis of PS and control rats and comparing phosphorylation (activation) levels of the MAPK isoforms p38 MAPK, ERK, and JNK by Western blotting (Figure 4C, Figure S6). Both native p38 MAPK and phosphorylated p38 MAPK (p-p38 MAPK) expression levels were significantly upregulated by both PS and β-EP administration, while ERK and JNK were unchanged on day 21, suggesting that p38 MAPK is a downstream mediator of β-EP−MOR-induced apoptosis. Subsequently, we found that expressions levels of MOR and proapoptotic proteins Bax and caspase 3 were increased both following stress and β-EP administration, while expression of anti-apoptotic Bcl-2 was reduced. Further, these effects were reversed by naloxone (Figure 4D). Immunohistochemistry of testicular sections also showed enhanced p38 MAPK, Bax, and caspase3, and reduced Bcl-2 in LCs (Figure S4). Collectively, these results indicate that β-EP induces LC apoptosis via activation of MOR−p38 MAPK signaling.

### 3.5. β-EP Promotes TM3 Cell Apoptosis through the p38 MAPK Pathway

**Figure 5:**
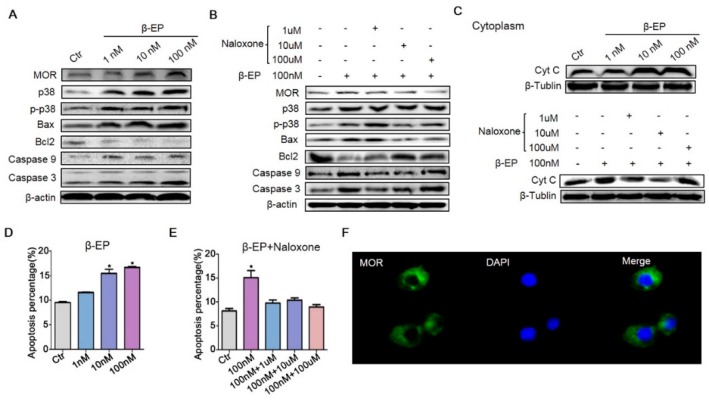
β-EP exposure induces apoptosis of TM3 cells. (**A**) Elevated expression levels of MOR, p38 MAPK, Bax, Bcl-2, caspase 9, and caspase 3 in TM3 cells after β-EP treatment as evidenced by western blotting. (**B**) Reversal of these effects by naloxone. (**C**) Elevated Cyt C after β-EP treatment and reversal by naloxone. (**D**) Elevated apoptosis and protection by naloxone as measured by flow cytometry. (**E**) Elevated apoptosis after β-EP treatment and reversal by naloxone as measured by flow cytometry. (**F**) Immunofluorescent staining of MOR (green) and DAPI (blue) in TM3 cells.

To further clarify the molecular pathogenesis of PS-induced LC apoptosis, we examined the signaling pathways in vitro. Since there are no internationally certified rat LCs, we used mouse TM3 cells. Treatment with 1, 10, or 100 nM β-EP increased the expression levels of MOR, p38 MAPK, Bax, caspase 9, and caspase 3 (Figure 5A). Also in accord with findings in vivo, β-EP treatment induced TM3 cell apoptosis (Figure 5B,D, Figure S5D). In addition, cytoplasmic cytochrome C (Cyt C) was increased by β-EP, indicating activation of the mitochondrial apoptosis pathway (Figure 5C). All of these changes were reversed by naloxone co-treatment (Figure 5B,C,E), while naloxone treatment alone had no significant effect (Figure S5C), providing further evidence that LC apoptosis under stress is mediated by β-EP−MOR signaling. Proliferation of TM3 cells was also inhibited by β-EP treatment (Figure S5A), an effect likewise reversed by naloxone (Figure S5B). Immunofluorescence showed that MOR was expressed both on the cell membrane and cytoplasm (Figure 5F).

**Figure 6:**
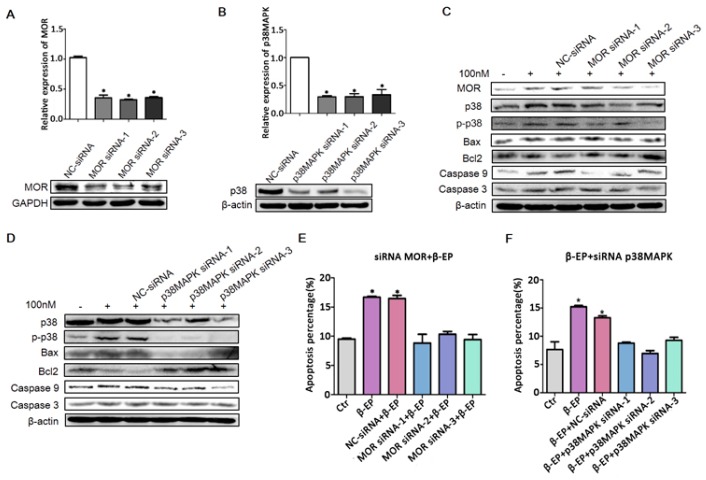
Contribution of the β-EP−MOR−p38 MAPK−Bax−caspase 3 pathway to LC apoptosis. Transfection of TM3 cells with si-MOR (**A**) or si-p38 MAPK (**B**) reduced the target gene expression level relative to a control gene (β-actin or GAPDH) as assessed by qRT-PCR and western blotting. (**C**,**D**) Elevated expression levels of MOR, p38 MAPK, Bax, Bcl-2, caspase 9, and caspase 3 in response to β-EP (100 nM) were reduced by si-MOR or si-p38 MAPK transfection but not by a negative control siRNA. Increasing TM3 cell apoptosis by β-EP was reversed by transfection with si-MOR (**E**) or si-p38 MAPK (**F**).

To confirm this mechanism of LC apoptosis, we transfected TM3 cells with siRNAs targeting MOR and p38 MAPK and examine effects on the response to β-EP. qRT-PCR and western blot analysis confirmed efficient interference efficiency (Figure 6A,B), and both MOR knockdown and p38 MAPK knockdown significantly reduced β-EP-evoked LC cell apoptosis (Figure 6C–F).

Collectively, the current study reveals that PS induces reproductive dysfunction through β-EP−MOR−p38 MAPK pathway activation and initiation of mitochondria-dependent apoptosis in LCs.

## 4. Discussion

It is generally accepted that PS contributes to numerous diseases by damaging the cardiovascular, immune, nervous, and (or) reproductive systems [27]. Both clinical and preclinical investigations have established PS as a major cause of male infertility [19], but the underlying molecular mechanisms are still largely unknown. Most animal models used for examining the effects of PS are established by physical stressors [28], which may have effects beyond those of PS. Therefore, a purely PS model was established, termed terrified sound PS [22,29,30]. PS substantially reduced sucrose intake. It is a sign of anhedonia associated with depression, accompanied by significant decreases in body, testis, and epididymis weight as well as serum reproductive hormone concentrations (GnRH, LH, FSH, and T). Moreover, sperm motility and concentration were significantly reduced, effects associated with accelerated apoptosis of testis LC cells. The testicular expression levels of ABP and BRDT proteins were also significantly downregulated by PS. Collectively, these findings indicate a substantial negative impact of PS on male rat reproductive function (Figure 1). The ABP protein is secreted by testicular Sertoli cells into the local extracellular space, reproductive ducts, and circulation, where it binds androgens. The ensuing reduction in free androgen concentration impairs spermatogenesis. Synthesis and secretion of ABP are regulated by FSH, and a significant reduction in ABP mRNA has been reported in patients with severe sperm damage [31]. FSH cognate receptor knockout mice also show lower levels of serum T and ABP, along with reduced fertility [32]. BRDT is specifically expressed in the testes in association with circular sperm nuclear chromatin, which is involved in chromatin recombination critical for maintenance of spermatogenesis [33]. BRDT inhibitors also blocked fertility in male mice [34]. Thus, ABP and BRDT can be used as markers of testicular reproductive dysfunction.

Psychological stress activates the HPA axis, resulting in excessive production and release of CRH, ACTH, and β-EP [3,35]. The increased β-EP secretion inhibits GnRH secretion by the hypothalamus, which in turn reduces FSH and LH section by the pituitary gland. The action of LH on the LCs to generate intratesticular T is essential for spermatogenesis [36]. The decrease in LH secretion then inhibits T secretion by LCs [37], thereby impairing spermatogenesis [9] and resulting in decreased sperm quantity and vigor [19,20]. Apoptosis occurs frequently, even during normal spermatogenesis. However, excessive apoptosis reduces sperm production, leading to infertility [38]. While PS appears to disrupt the normal regulation of both HPA and HPG axes, the specific mechanism are obscure. After long-term administration of β-EP to male rats, serum reproductive hormone levels and sperm quality were significantly reduced, and intracellular apoptosis was increased in the testis. Naloxone restored these abnormalities to control group levels, implicating β-EP−MOR signaling in PS-induced apoptosis in testis (Figure 2). Further, β-EP, its receptor MOR, phosphorylated p38 MAPK, Bax, and caspase 3 expression levels were upregulated by PS, and were co-localized to LCs (Figure 2, Figure 3 and Figure 4). These results revealed that PS-induced β-EP elevation, activation of MORs on LCs, ensuing p38 MAPK phosphorylation and finally lead to LCs apoptosis. To the best of our knowledge, this is the first study to report β-EP-induced apoptosis in LCs by PS.

The MAPKs has been linked to disturbances in spermatogenesis and dysfunction of germ cells and Sertoli cells, resulting in reduced semen quality and male reproductive dysfunction [39]. The current study implicates p38 MAPK-induced activation of the mitochondrial apoptosis pathway rather than the cell surface death receptor pathway. Apoptotic process during spermatogenesis would lead to defective sperm formation and potentially lead to infertility. The external agents including testicular toxins, heat stress and chemotherapeutic agents are stimuli for germ cell apoptosis in testicular tissue [40]. Previous study showed that p38 MAPK increased MAPK14 activation and involved in heat-induced sperm apoptosis in bovine through incubation at a hyperthermic (41 °C) temperature [41]. Activation of p38 MAPK under stress is a key event in apoptosis induction [42]. Antiapoptotic Bcl-2 levels decrease with rising p38 MAPK activity, indicating that p38 MAPK is the primary upstream signal controlling the balance between Bcl-2 and Bax expression in mitochondria [43,44,45]. Phosphorylation of p38 MAPK induces the release of Cyt C from mitochondria by inhibiting Bcl-2 and shifting the Bcl-2: Bax ratio, resulting in sequential activation of apoptotic proteases apof-1, caspase-9, and caspase-3 in germ cells [46]. Consistent with this mechanism, siRNA-mediated knockdown of MOR and p38 MAPK in LCs like TM3 cells decreased caspase 3 activity and apoptosis in response to β-EP. In addition, a study also reported that nicotine induces apoptosis in TM3 mouse LCs [47].

We demonstrate that the p38 MAPK signaling pathway is necessary for LC apoptosis under PS. However, p38 MAPK can be activated by a variety of upstream signals, control the expression of multiple transcription factors, and interact with other signaling pathways, so the detailed molecular pathway through which β-EP−MOR−p38 MAPK activation induces LC apoptosis remains to be determined. In addition, this study was only conducted in animal models—not with human patients’ samples—because it is difficult to collect human tissue for this kind of disease. Nonetheless, these findings underscore the promise of the β-EP−MOR−p38 MAPK signaling pathway as an important therapeutic basic for male reproductive disorders. The current study provided new content for the theoretical mechanism of male infertility caused by PS.

## Supplementary Materials

Supplementary Materials are available online at https://www.mdpi.com/2073-4409/8/10/1265/s1.
