# Supplementary material for: β-Endorphin Induction by Psychological Stress Promotes Leydig Cell Apoptosis through p38 MAPK Pathway in Male Rats
Source: Cells. 2019 Oct 16;8(10):1265. doi: 10.3390/cells8101265 (PMC6829611; doi:10.3390/cells8101265)
Supplement: Supplementary file 1 [file cells-08-01265-s001.pdf]

β-endorphin induction by psychological stress promotes Leydig cell apoptosis through p38 MAPK pathway in male rats

Xiaofan Xiong[#1,2], Lingyu Zhang[#1,2], Meiyang Fan[1,2], Lin Han[1,2], Qiuhua Wu[1,2], Siyuan Liu[1,2], Jiyu Miao[1,2], Liying Liu[2], Xiaofei Wang[2], Bo Guo[1,2], Dongdong Tong[1,2], Lei Ni[1], Juan Yang*[1,2], Chen Huang*[1,2]

Figure S1. The experimental procedures in this study. Psychological stress adopted a 'Terrified sound' recorded previously. Animal models were established through the psychological stress model and the intervention Group processing model. The animal models were established for 21 days, and the experimental samples were obtained on day 7, 14, and 21. Then, the extracted samples were used for reproductive related experiments. Finally, LCs were cultured with β-EP and naloxone intervention to clarify the molecular mechanism of apoptosis signal pathway promoted by β-EP.

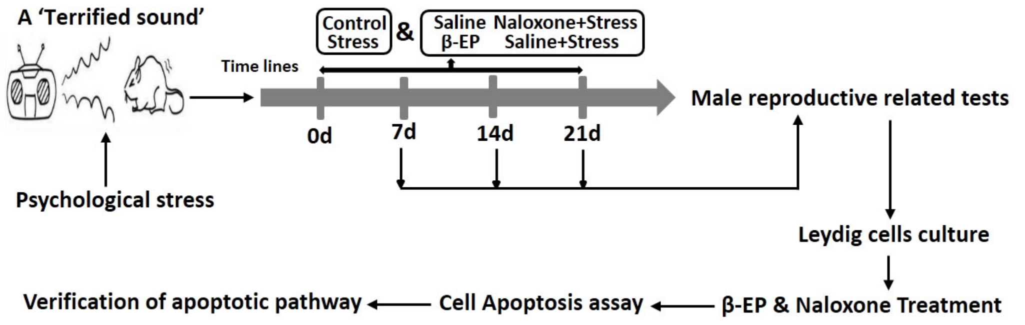

Figure S2: PS disrupts sex hormone regulation and reduces spermatogenic cells in the testes. Higher serum CRH (A) and ACTH (B) concentrations in PS rats vs. controls. (C) HE staining of testicular tissue sections after 21 days of PS compared to controls. Serum CRH (D), ACTH (E), and CORT (F) concentrations in rats treated with saline, β-EP, saline plus PS, or PS plus naloxone as indicated.

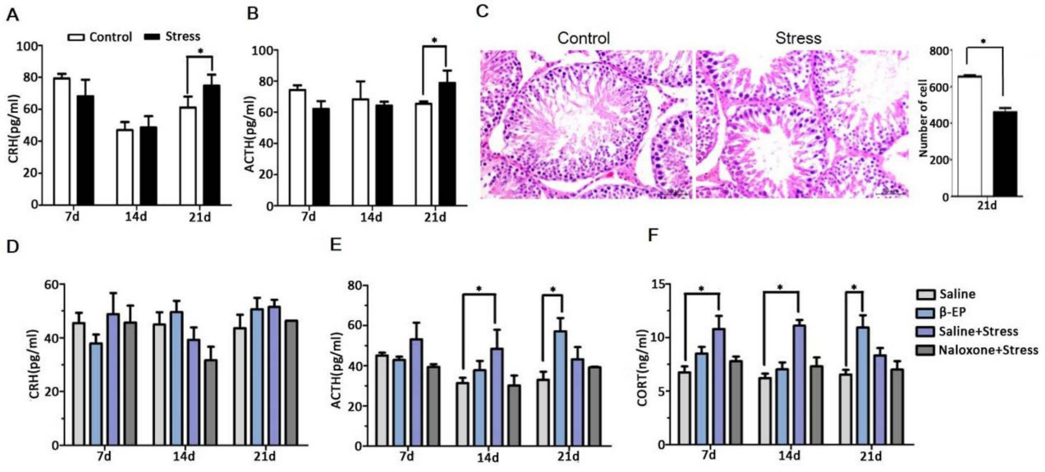

Figure S3: PS upregulates DOR and KOR expression levels in rat testes. (A1, A2) DOR and (B1, B2) KOR expressions in testicular tissue sections of PS rats vs.

controls on day 21 were detected by immunohistochemistry and evaluated by H-score. Bar = 50 μm. Positive cells are mainly distributed in LCs (red triangle) and the lumen of the seminiferous tubules (blue circle). (C) Western blot analysis of DOR and KOR expression levels in PS rat testis on day 21.

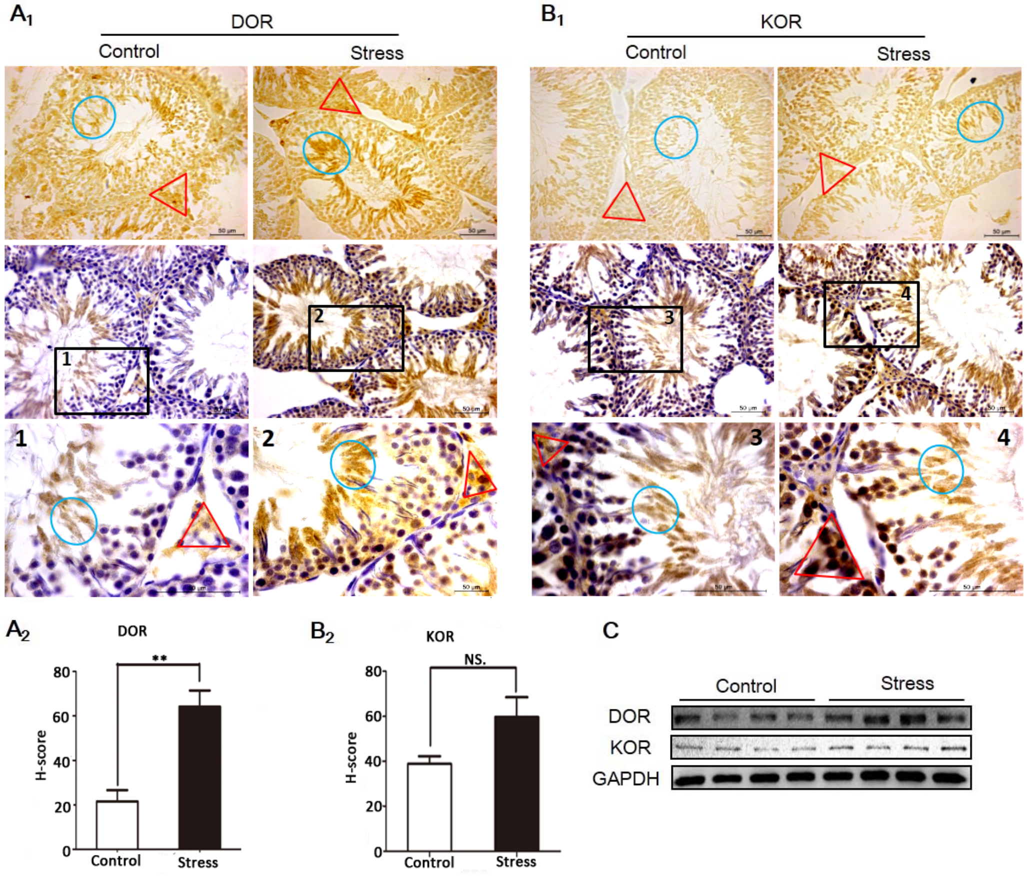

Figure S4: Expression of p38 MAPK, Bax, Bcl-2, and caspase 3 in testicular tissue sections from rats treated with saline, β-EP, saline plus PS, or PS plus naloxone were detected by immunochemistry, and evaluated byH-score. LCs were circled by red triangle.

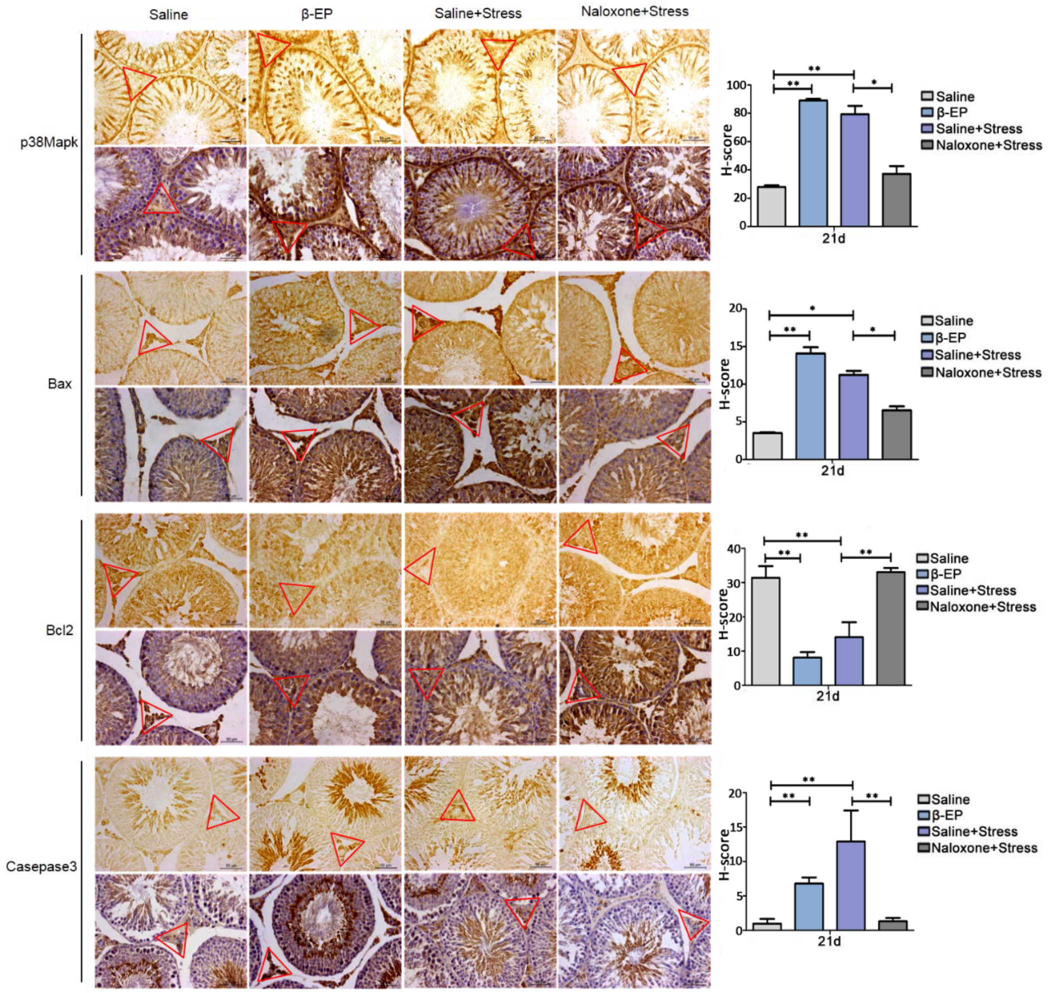

Figure S5: β-EP promoted apoptosis and inhibited proliferation of TM3 cells. (A) MTT assay of TM3 cells at 24, 48, and 72 h after treatment with β-EP. (B) MTT assay of TM3 cells at 24, 48, and 72 h after treatment with β-EP or β-EP plus naloxone. (C) Apoptosis assay of TM3 cells 48 h after treatment with naloxone alone. (D) Flow cytometric analysis of TM3 cell apoptosis 48 h after treatment with β-EP.

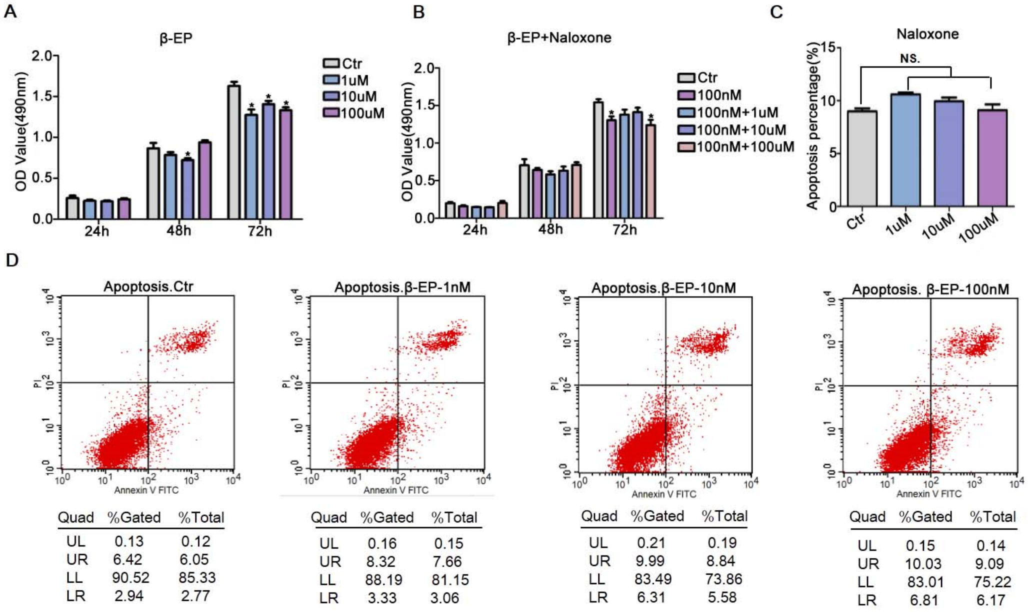

Figure S6: p38 MAPK involved in PS-induced apoptosis in testis. (A, B) (p-) p38 MAPK, (C, D) (p-) ERK, (E, F) (p-) JNK. All individual detections of all MAPKs proteins with its loading control on whole membrane but cut into two strips to incubate the antibodies separately, annotated with molecular weight, and quantification of blots by ImageJ software analysis are listed as following.

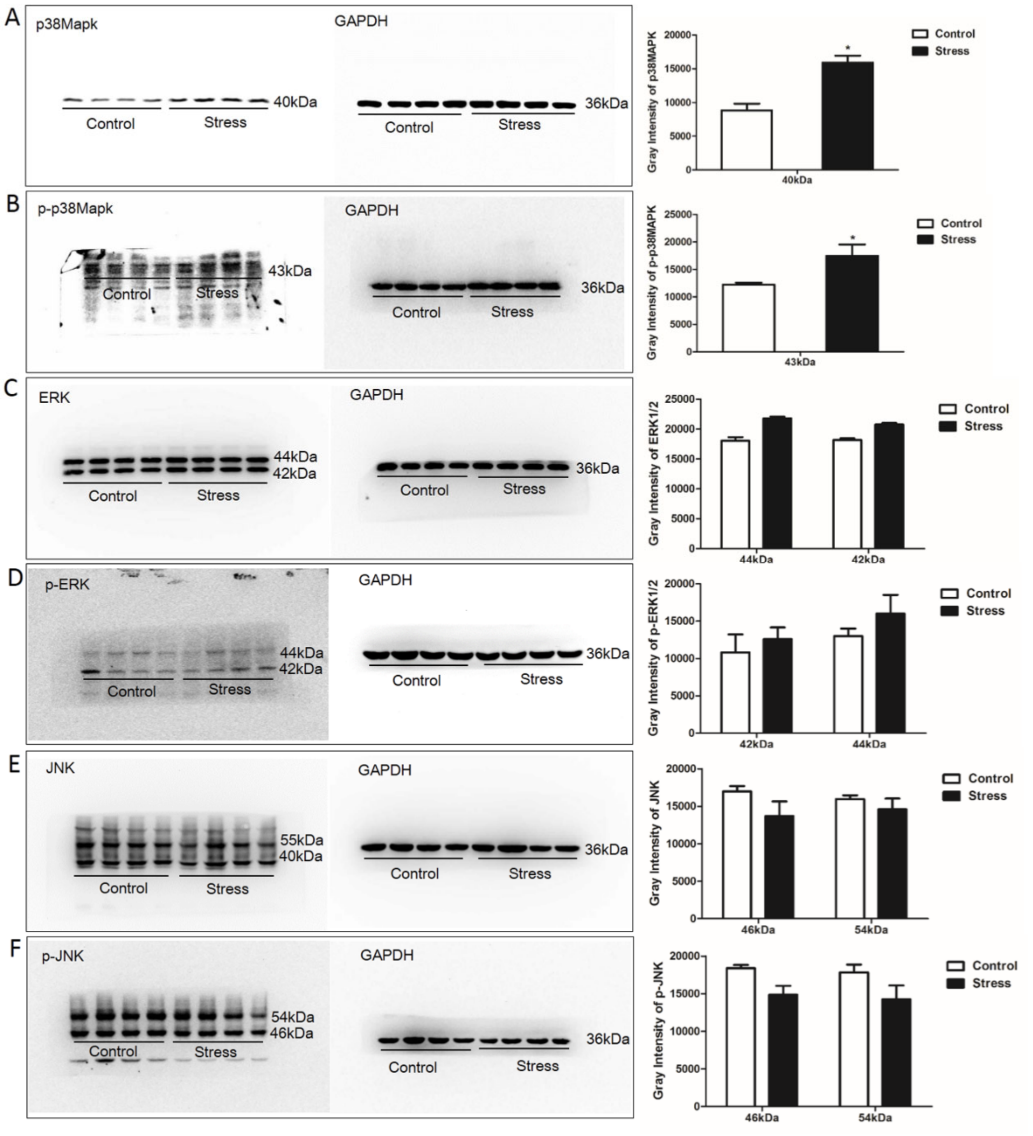

Table S1. Antibodies used in this work.

Antibody	Product number	Company	Molecular weight	Dilution
ABP antibody	bs-2410R	Bioss	40kDa	WB=1:500<br>IHC-P=1:400
BRDT antibody [C3]	GTX100201	GenTex	108 kDa	WB=1:1000
beta endorphin	bs-1195R	Bioss	4.5kDa	IHC-P=1:400
mu Opioid receptor	bs-3623R	Bioss	45kDa	WB=1:500<br>IHC-P=1:400
Delta Opioid Receptor	bs-3624R	Bioss	41kDa	WB=1:500<br>IHC-P=1:400
kappa Opioid receptor	bs-1094R	Bioss	43kDa	WB=1:500<br>IHC-P=1:400

P38MAPK(D13E1)XP	8690s	Cell Signaling Technology	40kDa	WB=1:1000<br>IHC-P=1:400
Phospho-p38 MAPK (Tyr 180/Tyr182) (D3F9)XP	4511s	Cell Signaling Technology	43kDa	WB=1:1000<br>IHC-P=1:800
JNK Antibody	66210-1-Ig	Proteintech	40kDa, 55 kDa	WB : 1:3000
Phospho-SAPK/JNK (Thr183/Tyr185)	#9255	Cell Signaling Technology	46kDa, 54 kDa	WB : 1:2000
p44/42 MAPK (Erk1/2) (137F5)	#4695	Cell Signaling Technology	42kDa, 44 kDa	WB : 1:1000
Phospho-p44/42 MAPK (Erk1/2) (Thr202/Tyr204)	#4370	Cell Signaling Technology	42kDa, 44 kDa	WB : 1:2000
Cytochrome C	66264-1-Ig	Proteintech	12 kDa	WB : 1:5000
Bax	50599-2-Ig	Proteintech	21 kDa	WB=1:2000<br>IHC : 1:50
Bcl-2	bs-0032R	Bioss	26kDa	WB=1:500<br>IHC=1:400
Caspase-9 (C9)	9508	Cell Signaling Technology	46kDa	WB=1:1000
Caspase 3	19677-1-AP	Proteintech	32 kDa	WB=1:1000<br>IHC : 1:100
β-Actin	sc-47778	Santa Cruz Biotechnology	42 kDa	WB=1:5000
GAPDH	60004-1-Ig	Proteintech	36 kDa	WB=1:5000

Table S2. Sequences of siRNAs used in this work.

Name	Sense(5’→3’)	Antisense(5’→3’)
NC-siRNA	UUCUCCGAACGUGUCACGUTT	ACGUGACACGUUCGGAGAATT
MOR siRNA-1:	CGUGGCCCUUUGGAAACAU	AUGUUUCCAAAGGGCCACG
MOR siRNA-2	CACCCCUCCACGGCUAAUA	UAUUAGCCGUGGAGGGGUG
MOR siRNA-3	CGAUGUUUUAGAGAGUUCU	AGAACUCUCUAAAACAUCG
p38MAPK siRNA-1:	GGUCACUGGAGGAAUUCAA	UUGAAUUCCUCCAGUGACC
p38MAPK siRNA-2:	GCUCAUUUUAAGACUCGUU	AACGAGUCUUAAAAUGAGC
p38MAPK siRNA-3:	GCAGGGACCUUCUCAUAGA	UCUAUGAGAAGGUCCCUGC

Table S3. Primer sequence used for qRT-PCR in this work.

Gene	Primer sequence	
	Forward(5’-3’)	Reverse(5’-3’)
MOR	CCAGGGAACATCAGCGACTG	GTTGCCATCAACGTGGGAC
p38 MAPK	TGACCCTTATGACCAGTCCTTT	GTCAGGCTCTTCCACTCATCTAT
GAPDH	GGTGAAGGTCGGTGTGAACG	CTCGCTCCTGGAAGATGGTG

Supplementary analysis method:

Counting workflow for the number of apoptotic cells.

1. Open the β-EP image of Figure 3B1 by “File/Open”, and change the image mode of color to

an 8-bit grayscale image in “Image/Type/8-bit” (as shown below).

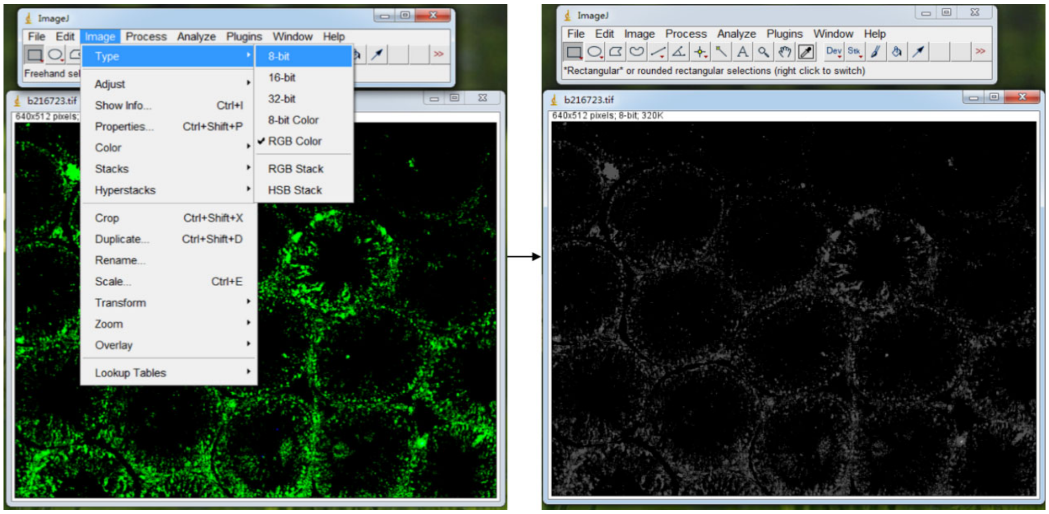

2. Increase the image contrast by “Image / Adjust / Brightness / Auto”.

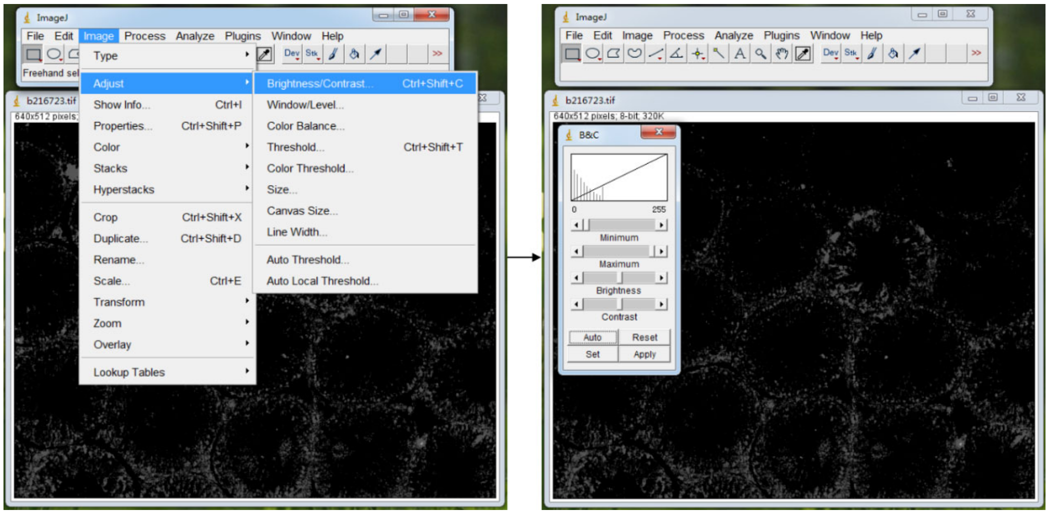

3. Use “Image/Adujust/Threshold”, and adjust the two sliders of the “Threshold” window to change the position of the red wire frame. The size range of the red wireframe determines the size range of the "particles" in the image. The range setting of “threshold” is the same for each group of images.

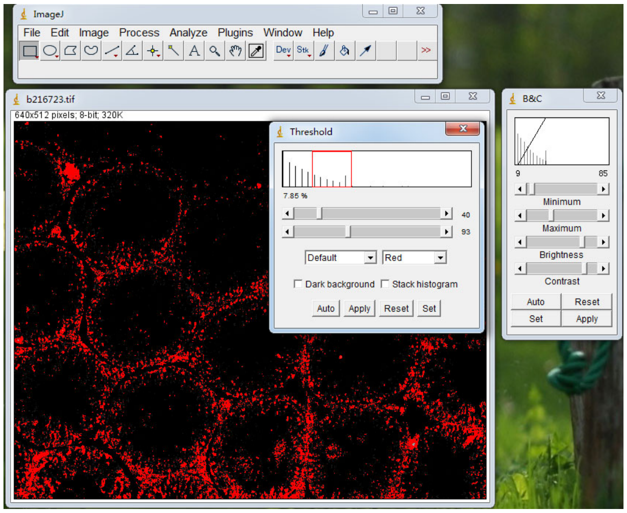

4. Enter the Analyze Particles window via "Analyse/Analyze Particales", set the minimum size of Particles to "50" here, select "Outlines" in "Show" to display the particles in the form of outer contours and number them, select "Summarize" and click "OK".

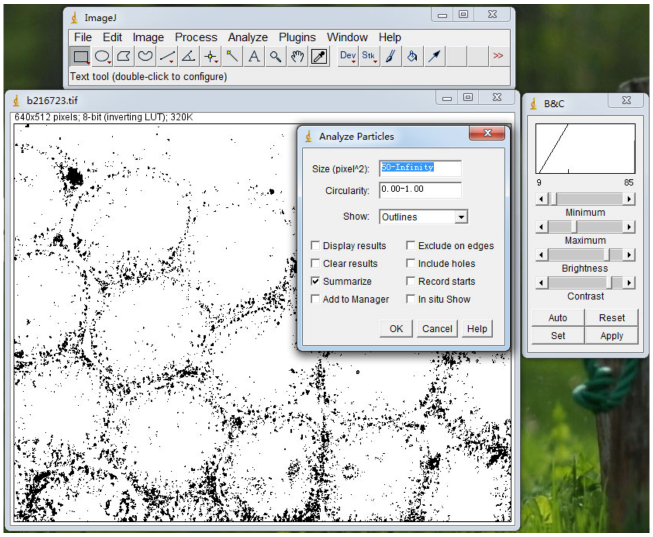

5. The result of the counting is displayed in the "Summary" window. As shown in the figure below, a total of 69 "particles" are counted. This is the number of apoptotic cells.

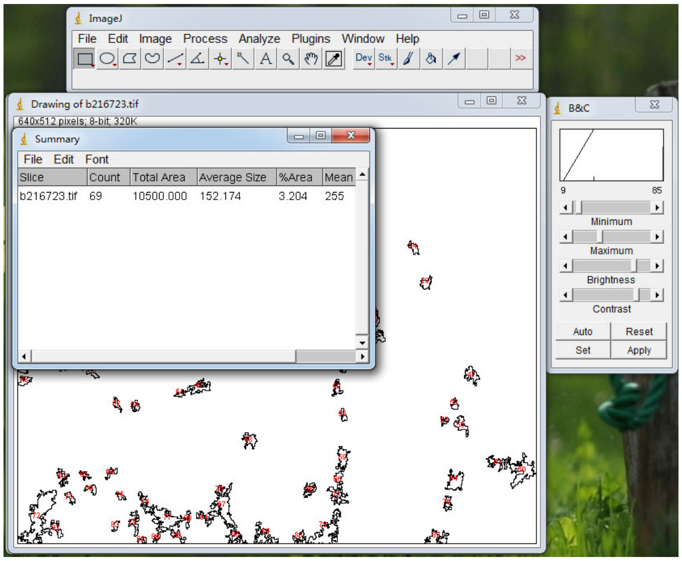
ImageJ
File Edit Image Process Analyze Plugins Window Help
Drawing of b216723.tif
640x512 pixels; 8-bit; 320K
Summary
File Edit Font
Slice Count Total Area Average Size %Area Mean
b216723.tif 69 10500.000 152.174 3.204 255
B&C
9
85
Minimum
Maximum
Brightness
Contrast
Auto
Reset
Set
Apply
